# Glucosamine and Chondroitin Sulfate: Is There Any Scientific Evidence for Their Effectiveness as Disease-Modifying Drugs in Knee Osteoarthritis Preclinical Studies?—A Systematic Review from 2000 to 2021

**DOI:** 10.3390/ani11061608

**Published:** 2021-05-29

**Authors:** Silvia Fernández-Martín, Antonio González-Cantalapiedra, Fernando Muñoz, Mario García-González, María Permuy, Mónica López-Peña

**Affiliations:** 1 Anatomy, Animal Production and Veterinary Clinical Sciences Department, Veterinary Faculty, Campus Universitario s/n, Universidade de Santiago de Compostela, 27002 Lugo, Spain; antonio.cantalapiedra@usc.es (A.G.-C.); fernandom.munoz@usc.es (F.M.); mariog.gonzalez@usc.es (M.G.-G.); monica.lopez@usc.es (M.L.-P.); 2 Ibonelab S.L., Laboratory of Biomaterials, Avda. da Coruña, 500 (CEI-NODUS), 27003 Lugo, Spain; permuy@ibonelab.com

**Keywords:** animal models, biochemical markers, cartilage, chondroitin sulfate, glucosamine, nutraceuticals, osteoarthritis

## Abstract

**Simple Summary:**

Osteoarthritis is the most common progressive joint disease diagnosed in companion animals and its management continues to be a significant challenge. Nutraceuticals have been widely investigated over the years in the treatment of osteoarthritis and among them, glucosamine and chondroitin sulfate treatments are probably the most common therapies used in veterinary management. However, heterogeneous results were obtained among animal studies and the evidence of their efficacy is still controversial. Animal models have a crucial role in studying the histological changes and evaluating the therapy efficacy of different drugs. Consequently, we consider it may be of interest to evaluate the effectiveness of the most representative nutraceuticals in experimental animal studies of osteoarthritis. In this systematic review, we found a large inconsistency among the experimental protocols, but a positive cartilage response and biochemical modulation were observed in half of the evaluated articles, mainly associated with pre-emptive administrations and with some therapies’ combinations. Even though some of these results were promising, additional data are needed to draw solid conclusions, and further studies evaluating their efficacy in the long term and focusing on other synovial components may be needed to clarify their function.

**Abstract:**

Glucosamine and chondroitin sulfate have been proposed due to their physiological and functional benefits in the management of osteoarthritis in companion animals. However, the scientific evidence for their use is still controversial. The purpose of this review was to critically elucidate the efficacy of these nutraceutical therapies in delaying the progression of osteoarthritis, evaluating their impact on the synovial knee joint tissues and biochemical markers in preclinical studies by systematically reviewing the last two decades of peer-reviewed publications on experimental osteoarthritis. Three databases (PubMed, Scopus and, Web of Science) were screened for eligible studies. Twenty-two articles were included in the review. Preclinical studies showed a great heterogeneity among the experimental designs and their outcomes. Generally, the evaluated nutraceuticals, alone or in combination, did not seem to prevent the subchondral bone changes, the synovial inflammation or the osteophyte formation. However, further experimental studies may be needed to evaluate their effect at those levels. Regarding the cartilage status and biomarkers, positive responses were identified in approximately half of the evaluated articles. Furthermore, beneficial effects were associated with the pre-emptive administrations, higher doses and, multimodality approaches with some combined therapies. However, additional studies in the long term and with good quality and systematic design are required.

## 1. Introduction

Osteoarthritis (OA) is a heterogeneous chronic disease that involves all tissues in the synovial joints. It is usually characterized by progressive cartilage damage, subchondral bone changes, osteophyte formation, synovial inflammation and the secretion of inflammatory mediators [1,2]. At present, it is most common progressive joint disease diagnosed in companion animals and its management continues to be a significant challenge [3]. Lameness, stiffness and chronic pain resulting from the OA pathology have a negative impact on the quality of life of the affected animals [4]. Additionally, OA pain is frequently mishandled in animals, and consequently, some clinical cases may result in premature euthanasia [5].

At present, there is hardly any accurate epidemiological data available of this disease in the different animal species [6]. In sport horses, OA is one of the most prevalent and disabling diseases in sport horses, fundamentally affecting the metacarpophalangeal joint and causing chronic and painful lameness as well as an important economic loss in the equine industry [7]. Furthermore, a recent study has reported a noteworthy prevalence of cervical OA in jumping horses. More specifically, a moderate to severe OA was observed at C6-7 in 25% of the studied population [8]. In dogs, OA is highly prevalent with reports of around 20% of the canine population over a year-old [9]. Nevertheless, subsequent studies reported lower values, as observed by Anderson et al. [3] and O’Neill et al. [10], who estimated an OA prevalence of 2.5% or 6.6% in primary-care practices in the UK. Generally, large-breed dogs developed initial and more severe clinical signs of OA [4]. However, early symptoms may be overlooked by the owner or considered normal, thus the joint disease is usually diagnosed at a later stage [3]. In cats, it is a very common joint disease, especially in older cats. In relation to this, a previous study reported an OA prevalence of around 61% at over 6 years of age [11]. OA in cats seems to be related to behavioural changes such as decreased mobility and less grooming [11]. However, it is important to highlight the underdiagnosis of the disease associated with the lack of signs such as lameness and a lower radiographic identification. This is in addition to its difficult physical examination by clinicians [12]. Furthermore, the treatment of OA is a major challenge in this specie, related to reduced availability of drugs as well as increased adverse effects and complications [13].

For many years, the available therapeutic options for OA management were focused on inflammation relief and pain control and were basically restricted to the use of non-steroidal anti-inflammatory drugs and analgesics. However, their chronic administration was limited by their deleterious systemic side effects [6]. Currently, there is no ideal drug capable to reverse or stop the progression of the OA and for that purpose, numerous therapeutic agents have been widely researched for their potential role in targeting the underlying pathology of OA with various levels of efficacy [14]. Nutraceuticals, also classically called chondroprotectors, have been widely analysed over the years in the treatment of OA in companion animals. Among them, glucosamine and chondroitin sulfate treatments are probably the most commonly used in the veterinary management of OA [4]. These dietary supplements have been proposed to promote the cartilage and periarticular bone health status [15] and their effectiveness in the OA progression has been thoroughly tested in experimental research. However, heterogeneous results were obtained in different animal studies and their function as disease-modifying drugs is still controversial. Some published clinical trials in dogs treated with glucosamine and chondroitin sulfate, reported positive clinical effects with significant pain relief [16], whereas in other publications, no significant differences were found between treated and untreated dogs [17,18].

At present, there are multiple reviews focusing on the effects of dietary supplements in clinical signs of companion animal OA, as an attempt to clarify their effectiveness in the OA management [4,19,20,21]. In this context, a previous literature review studied some nutraceutical effects in different OA animal models [22]. However, in that case, they fundamentally focused on determining the most suitable animal model to examine the potential beneficial roles of different nutraceuticals such as vitamins, avocado and soybean, polyphenols and glycosaminoglycans, among others. To the best of our knowledge, no current systematic reviews have evaluated their impact on the structural OA changes in animal models. Preclinical animal models offer a great opportunity to better understand the pathophysiology of the OA and to evaluate the therapeutic response [23]. Consequently, we consider it may be of interest to review, synthesise and evaluate the effectiveness of the most representative nutraceuticals in different animal model studies, given the available scientific data.

The purpose of this review was to critically elucidate the efficacy of glucosamine and chondroitin sulfate therapies in delaying the progression of OA, evaluating their impact on the synovial knee joint tissues and biochemical markers in preclinical studies by systematically reviewing the last two decades of peer-reviewed publications on experimental OA.

## 2. Materials and Methods

### 2.1. Protocol and Search Strategy

This systematic review followed the Preferred Reporting Items for Systematic Reviews and Meta-Analyses (PRISMA) guidelines [24]. The literature search was performed using the following online scientific databases: PubMed, Web of Science and Scopus. The studies were identified using combinations of the following terms: “osteoarthritis”, “animal models”, “glucosamine” and/or “chondroitin sulfate” as keywords.

The inclusion criteria were as follows:

- Experimental preclinical studies in animal models of OA which focused on the structural effect of glucosamine and chondroitin sulfate on the knee synovial joint tissues and biochemical markers.
- Studies that included outcome measures by using gross, histologic, histomorphometric, biochemical and/or imaging techniques.
- Articles written in English language.
- Studies published in international peer-reviewed journals between 2000 and February 2021.

The exclusion criteria were articles written in other languages, reviews, abstracts, book chapters, in vitro studies and clinical human or animal trials. Furthermore, articles that did not include an OA animal model, with intraarticular administrations of therapies, or reports in which the joint of interest was not the knee, were also excluded.

### 2.2. Study Selection and Data Extraction

The titles, abstracts and the full text of the articles, identified by Internet searches, were screened by two reviewers (S.F.-M. and A.G.-C.). The eligible papers were carefully read, and the following data were extracted: author and year of publication, animal model (breed, species, gender, age and number of animals), OA model according with its cause, nutraceuticals therapy including type, dosage, frequency and the administration route, baseline, time of sacrifice, outcome measures and main results.

Subsequently, we independently evaluated the main outcomes determined in the included articles based on the type of nutraceuticals studied and their structural effects on the cartilage, subchondral bone and synovial membrane. Additionally, their influence on the osteophyte formation and biomarkers fluctuations was taken into account. We classified the outcomes as positive effect (+), negative effect or no effect (−) and unclear effect or no significant effect (?). In addition, we marked as not included (x) when the parameters were not evaluated in the study. Regarding the initial administration of the therapies, we classified them as pre-emptive therapies if they were administered before OA induction, early therapies when they were administered between OA induction and 14 days post-OA induction, and delayed therapies if the baseline was more than 14 days post-OA induction.

In addition, the studies were classified based on the therapy duration, into short-term therapies (≤8 weeks), intermediate-term therapies (>8 to <24 weeks) and long-term therapies (≥24 weeks). In the studies where different therapy durations were included, we selected the longest term.

### 2.3. Quality Assessments and Risk of Bias

The quality and risk-of-bias assessments were performed by two independent authors (S.F.-M. and A.G.-C.), and any discrepancies were resolved by team consensus with all the authors. To assess the quality of the animal studies, we analysed all the included manuscripts using the updated guidelines for reporting animal research: the ARRIVE guidelines (Animal Research: Reporting of In Vivo Experiments) [25]. More specifically, we used the “Compliance Questionnaire” in order to evaluate whether the manuscripts complied with the ARRIVE Essential 10: Study design, sample size, inclusion and exclusion criteria, randomisation, blinding, outcome measures, statistical methods, experimental animals, experimental procedures and results. We checked each of the 10 items and assigned the category of “reported” if the item was completely reported, “not reported” if it was not reported and “unclear” if it partially reported or if insufficient details were provided.

The risk of bias was assessed using the Systematic Review Centre for Laboratory animal Experimentation (SYRCLE) tool for animal studies in order to assign a judgement of low, high or unclear risk of bias to each of the 10 items included in the checklist: sequence generation, baseline characteristics, allocation concealment, random housing, blinding caregivers and/or investigator, random outcome assessment, blinding outcome assessor, incomplete outcome data, selective outcome reporting and other sources of bias [26].

## 3. Results

### 3.1. Study Selection and Characteristics

**Figure 1:**
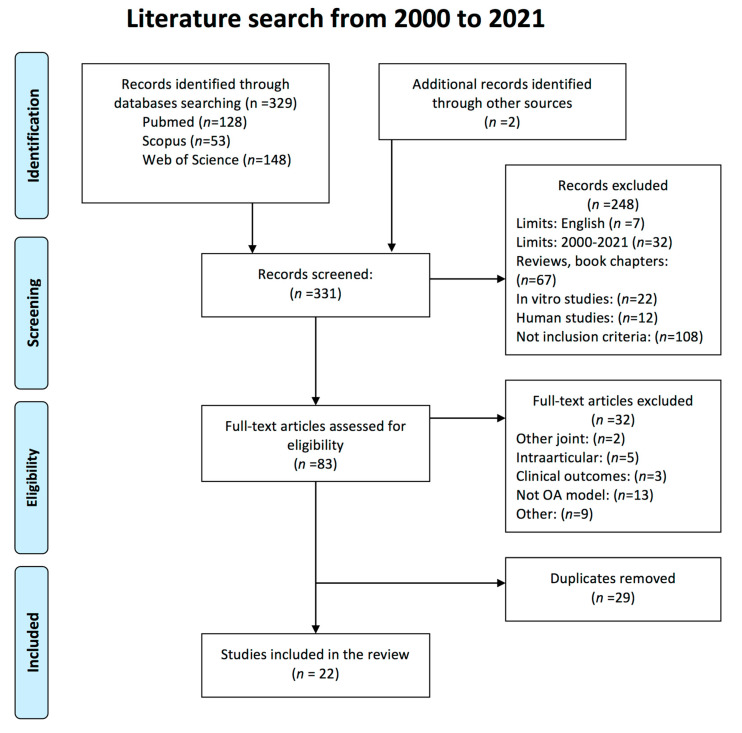
Search strategy according to Preferred Reporting Items for Systematic Reviews and Meta-Analyses (PRISMA) guidelines of preclinical animal studies of the effect of glucosamine and chondroitin sulfate in knee osteoarthritis.

The initial literature search resulted in 329 potentially eligible articles. 128 publications were obtained using PubMed, 53 using Scopus and 148 using Web of Science. Additionally, 2 papers were identified through ResearchGate network and were also included. After reading the title and the abstract, 248 records were excluded and the remaining publications (*n* = 83) were checked in full-text. 32 of them were excluded after determining that they did not meet the inclusion criteria, 29 duplicates were removed and finally, a total of 22 studies were included in the present systematic review. The publications dated from 2005 to 2019 and were published in 19 different journals. The flow chart of the selected articles is shown in Figure 1.

The main characteristics and results of the 22 included preclinical studies are shown in Table 1. Most of the preclinical studies were performed in rats (11 out of 22; 50%), followed by rabbits (9 out of 22; 40%), mice (1 out of 22; 5%) and guinea pigs (1 out of 22; 5%). Regarding the animal model selected, in most studies OA was surgically induced by anterior cruciate ligament transection (ACLT) and/or medial meniscectomy (MMT) (16 out of 22; 73%). Two studies in rats [27,28] and the one which included the mice model [29] used intraarticular injections to chemically induce OA. Only two studies, one involving rabbits [30] and another involving rats [31], used physically immobilisation to induce the OA. Lastly, only one study in guinea pigs was included as a spontaneous model of OA [32]. 

### 3.2. Synthesis of the Main Outcomes of the Effect of Glucosamine and/or Chondroitin Sulfate

The analysed studies were classified based on the therapy evaluated and its effects on the synovial joint tissues, osteophyte development and biochemical markers (Table 2). In the 22 studies included in this review, 26 nutraceutical effects have been evaluated, distributed as follows: Glucosamine sulfate (GS) (*n =* 6), glucosamine hydrochloride (GH) (*n =* 8), chondroitin sulfate (CS) (*n =* 5), CS+GH (*n =* 3) and CS+GS (*n =* 4).

Most of the publications analysed the glucosamine effect (*n* = 14) in its hydrochloride (*n* = 8) or sulfate (*n* = 6) form. Less number of publications analysed the effect of the chondroitin sulfate, administered either alone (*n* = 5) or in combination with glucosamine sulfate (*n* = 4) or glucosamine hydrochloride (*n* = 3). Additionally, it should be noted that one study included the evaluation of glucosamine sulfate and chondroitin sulfate separately [36], another the glucosamine sulfate and the glucosamine hydrochloride [29], another the effect of the glucosamine hydrochloride and the chondroitin sulfate [32] and another the combination of chondroitin sulfate plus glucosamine hydrochloride against chondroitin sulfate plus glucosamine sulfate [35]. Consequently, as we explained before, within the 22 studies included in the present systematic review, 26 evaluations of the nutraceutical effect alone or in combination were carried out.

Regarding the parameters evaluated, the cartilage response is by far the most assessed, being included in 25 evaluations out of 26. Positive chondroprotective effects were identified in approximately half of the evaluations (14 out of 25; 56%). In the individual analyses, the results were as follows: glucosamine sulfate (4 out of 6; 67%), glucosamine hydrochloride (4 out of 8; 50%), chondroitin sulfate (3 out of 5; 60%); chondroitin sulfate plus glucosamine hydrochloride (1 out of 3; 33%) and chondroitin sulfate plus glucosamine sulfate (2 out of 3; 67%). The biochemical markers of OA were the second most studied parameter in this systematic review and was included in 20 out of 26 therapy assessments. Nutraceuticals showed a positive effect in 13 of them (13 out of 20; 65%). Specifically, in terms of glucosamine therapies, we identified positive responses in 4 out of 5 (80%) sulfate formulations and in 4 out of 6 (67%) hydrochloride ones. With respect to chondroitin sulfate, 3 out of 5 publications included biomarker evaluations and, in this case, all of them showed fewer biochemical alterations in the treated groups. The subchondral bone changes were determined in 8 out of the total number of included evaluations, identifying beneficial effects in only two of the publications (2 out of 8; 25%) [28,38]. The synovial inflammation was evaluated in 7 studies, showing supressed synovitis in only one of them (1 out of 7; 14%) [46]. Finally, the osteophyte development was evaluated in 3 studies, but only in one of them a reduced osteophyte formation was observed after glucosamine hydrochloride administration [29].

### 3.3. Therapy Duration and Initial Administration at Baseline

The nutraceutical therapy periods were shown in Table 3. The majority of the preclinical studies included in the systematic review were based on short-term therapies (*n* = 15). Intermediate-term therapies were employed in 6 of the selected publications, with nutraceutical treatment periods lasting 8–24 weeks. Lastly, the review only included a publication which studied the long-term therapy response in a guinea pig spontaneous OA model [32].

Regarding the therapy timing initiation in relation to OA induction, most of the studies applied early therapy administrations, up to only 14 days post experimental OA induction. 5 out of 22 articles studied the effect of these therapies in delayed administrations (>14 days post OA induction) and finally, 4 studies focused on the pre-emptive responses (before OA induction) (Table 2). The chondroprotective effect was observed in 7 out of the 13 publications with early treatment administrations, making up for 54%, 3 out of 5 publications with delayed initial treatments, corresponding to 60% and finally, 3 out of 4 pre-emptive protocols making up for 75%.

### 3.4. Quality and Risk-of-Bias Assessments

**Figure 2:**
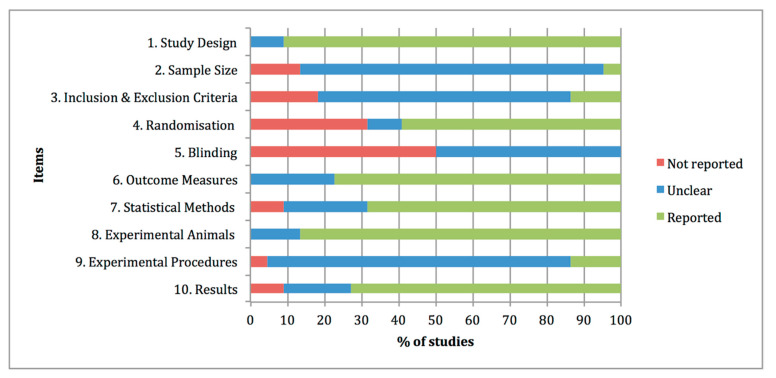
Quality assessments of the 22 preclinical studies included in the systematic review based on the Essential 10 items of the ARRIVE guidelines 2.0. Values are expressed by frequencies (%).

The quality assessments of the preclinical studies based on the essential 10 items of the ARRIVE guidelines were summarised in Figure 2. The individual analysis of the manuscripts showed that at items 4 “Randomisation” and 5 “Blinding”, information was not adequately reported in 32% and 50% of the studies, respectively. By contrast, at items 1 “Study design”, 6 “Outcome measures”, 7 “Statistical methods”, 8 “Experimental animals” and 10 “Results”, adequate and clear information was reported in the experimental studies, with percentages of 91%, 77%, 68%, 86% and 73% of the studies. Other items, such as 2 “Sample size”, 3 “Inclusion and exclusion criteria” and 9 “Experimental procedures” were graded as unclear with percentages of 82%, 68% and 82% of the studies, due to partially reported or insufficient experimental details provided in the studies.

**Figure 3:**
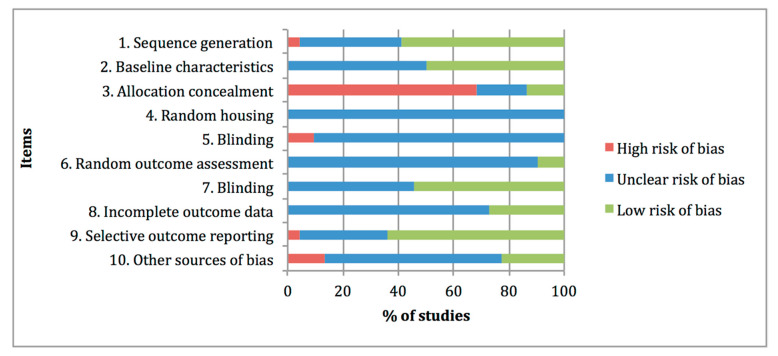
Risk of bias distribution graph of the 22 preclinical studies included in the systematic review according to SYRCLE tool. Values are expressed by frequencies (%).

Figure 3 summarises the risk-of-bias distribution results obtained with the SYRCLE tool. The lower risk of bias was observed at items 1 “Sequence generation, 7 “Blinding of outcome assessor” and 9 “Selective outcome reporting”, with percentages of 59%, 55% and 64%, respectively. The higher risk of bias was assigned at item 3 “Allocation concealment” with a percentage of 68%, whereas high frequencies of unclear risk of bias ratings were assigned at items 4 “Random housing”, 5 “Blinding of caregivers”, 6 “Random outcome assessment”, 8 “Incomplete data outcome” and 10 “Other sources of bias”, with percentages of 100%, 91%, 91%, 73% and 63%, respectively.

## 4. Discussion

The aim of this systematic review was to examine the effect of glucosamine and chondroitin sulfate treatments in the synovial knee joint tissues and specific biomarkers of the osteoarthritic preclinical studies. A total of 22 studies with 3 different types of nutraceuticals: glucosamine sulfate, glucosamine hydrochloride and chondroitin sulfate, administered alone or in different combinations, were meticulously analysed in order to elucidate their direct influence on the main structural and biochemical elements in the OA joints.

Taking into account the experimental animal model, in the studies considered for this review, the most commonly used species were rats and rabbits, whereas only one study employed mouse as animal models [29]. These findings were different from those observed by other OA preclinical research, where mouse models constituted the majority of the included studies [49,50]. In regards to OA induction is concerned, in agreement with other publications, surgically induced models were one of the most selected based on the rapid OA induction, repeatability and lower costs [23,51]. Regarding spontaneous models, a slower disease progression was observed and therefore it seems closer to what naturally occurs in primary osteoarthritic disease [52]. However this review included only one study of spontaneous model of OA in guinea pigs [32].

Generally, in this systematic review we found a large inconsistency among the experimental nutraceutical protocols. As an attempt to reduce the variability among studies, we excluded the articles in which animals received intra-articular therapy injections [53,54,55,56,57]. Even though according to the records screened, these local therapies demonstrated a positive chondroprotective effect and anti-inflammatory activity, we decided to evaluate other administrations routes, such as oral and intraperitoneal, in order to analyse the systemic and non-local effects of glucosamine and chondroitin sulfate.

With respect to the therapeutic regimen, there are also notable differences among studies, both in the frequency and in the dosage administered. In this regard, 2 out of 22 articles included in this review examined the efficacy of chondroitin sulfate [27] and glucosamine sulfate [41] at different doses, and both of them concluded that they seemed to reduce the cartilage changes and biomarker alterations in a dose-dependent manner.

Additionally, some articles analysed the potential chondroprotective effect of different nutraceutical combinations. Furthermore, the combination of nutraceutical and other therapies and drugs was also investigated. In this context beneficial effects were observed in the association of glucosamine and risedronate [31,36], glucosamine and fish collagen peptide [37] and glucosamine hydrochloride and chondroitin sulfate plus fursultiamine, where only the combined group with the addition of the vitamin showed a significant chondroprotective effect [39]. Furthermore, in one study using an OA experimental model in rats, enhanced responses were observed with the association of glucosamine sulfate, chondroitin sulfate and photobiomodulation [43].

Regarding the nutraceutical combinations between glucosamine and chondroitin therapies evaluated in this review, Silva et al. [48] observed that the association of glucosamine sulfate and chondroitin sulfate, rather than isolated glucosamine, significantly reduced the joint pain and prevented the cartilage histology alterations. Likewise, Terencio et al. [44] demonstrated the chondroprotective effects of the combination chondroitin sulfate-glucosamine, as well as reduced inflammatory mediator levels. By contrast, Roman-Blas et al. [35] did not find any beneficial effects in the combined therapy with chondroitin sulfate plus glucosamine sulfate or hydrochloride. Regarding this point, a study which used a chemically induced murine model, focused on examining the effect of glucosamine on cartilage degradation and bone resorption, comparing two different pharmacological forms, sulfate and hydrochloride; the results showed less histologic effectiveness in the sulfate form when both were administered under the same conditions [29]. It is important to point out that some authors suggested that glucosamine hydrochloride had poorer bioavailability and less beneficial effect in relieving clinical OA symptoms [21]. In our review, slightly higher chondroprotective effects were determined in the glucosamine sulfate studies comparing to the studies that included the glucosamine hydrochloride formulation (67% vs. 50%, respectively). Furthermore, fewer biochemical alterations were found in the glucosamine sulfate administration compared to the hydrochloride ones (80% vs. 67%). Nevertheless, in a previous review on the use of glucosamine in the management of human OA, the authors determined that, due to the heterogeneous effects observed in the available research studies, concluding which formulation could be more effective continues to be extremely difficult [58].

Another point of interest in the experimental design is the therapy timing initiation in relation to the OA induction. As previously described, the articles included in this review were grouped into three distinct protocols, pre-emptive, early and delayed administrations. Among these studies, the highest chondroprotective effects were determined in the pre-emptive therapies, followed by the delayed ones, which showed slightly positive higher values than the studies with early administrations. These findings were slightly different to those observed in a recently published systematic review about the effect of bisphosphonates therapies in OA preclinical studies [59], where an obvious time-dependent efficacy on cartilage status was determined, showing better chondroprotective effects in pre-emptive and early therapy initiations and greater cartilage damage in the delayed ones. In our opinion, the positive values observed in the delayed administrations could be associated with an inadequate selection of the period of time determined, given that it can be established as early as 14 days after de OA experimental induction and longer periods of time may be required.

In terms of duration, attention is drawn to the lack of evidence in long-term therapy, identifying only 1 out of 22 included publications, in which glucosamine hydrochloride and chondroitin sulfate were evaluated at 8, 12 and 18 months, showing reduced cartilage degeneration and biomarker alterations in both treated groups, the animal group treated with glucosamine hydrochloride and the group treated with chondroitin sulfate [32]. In this context, it is important to highlight that the histological and biochemical response after long-term nutraceutical administration is basically unpredictable. The initial positive response identified in some studies may not be sustained for long periods of time. However, the opposite is also possible, and a longer duration of treatment period may be necessary to observe a beneficial effect in the synovial joint. Therefore, additional preclinical studies in OA research evaluating the effect of dietary supplements in the long term are required [19,59].

Overall, in this systematic review, we observed a high variability among the experimental designs. Consequently, making an accurate assessment of how glucosamine and chondroitin sulfate affect the OA progression continues to be a challenge. In general terms, the evaluated nutraceuticals, alone or in combination, did not seem to prevent the subchondral bone changes, the synovial inflammation or the osteophyte formation, showing poor positive responses. Nevertheless, it is true that only a few of the publications included evaluations at those levels. Cartilage continues to be the primary focus in OA research and in this sense, positive chondroprotective effects were identified in approximately half of the publications, the studies of glucosamine sulfate and the combination of chondroitin sulfate plus glucosamine sulfate showing the most promising results. There is also increasing attention on the research of biochemical markers. As it could be observed in this study, they were the second more assessed parameter. In this context, a positive response was identified in more than half of the evaluations included in this review.

Regarding the risk of bias and the quality assessments of the articles included in this review, they were similar to the previous studies [49,59]. There are essential details about the experimental design which continue to be poorly reported in the studies, such as the sample size calculation, which was only reported in one of the manuscripts [33]. Inclusion and exclusion criteria were also badly or incompletely reported in most of the studies as well as blinding the experimental details. More specifically, half of the studies did not report the information and the other half only specified it in the outcome assessment stage, but not in the experimental and treatment administration stages. In addition, the information concerning the acclimatisation period of the animals, the housing and husbandry was also insufficient. In this sense, a previous research evaluated the adherence to the ARRIVE checklist in 236 papers between 2009 and 2015, and unexpectedly none of the evaluated manuscripts fully reported 100% of the items [60]. Consequently, the improvement of the research report in animal experimentation continues to be an essential task at present [25].

To conclude, OA management in companion animals continues to be a challenge in veterinary medicine. As we exposed in this review, glucosamine and chondroitin sulfate seems to provide chondroprotective effects and less inflammatory biochemical response in approximately half of the evaluations. However, these effects are inconsistent between the clinical and the preclinical studies. One explanation may be related to the great variety of histological scoring evaluations, the potential assessor’s subjectivity and the possibility of intra- and inter-observer variations [61]. Moreover, as these therapies have a slow onset of action, long-term administrations should be required to clarify their effectiveness. Additionally, a possible caregiver placebo effect may explain some of the beneficial responses observed in clinical trials with dogs [17,62]. For all these reasons, the use of glucosamine and chondroitin sulfate should be an individual veterinary/owner decision, reached by thoroughly evaluating each particular clinical case and its symptomatic response.

## 5. Conclusions

In this systematic review we found a large inconsistency among the experimental nutraceutical protocols and the outcomes of the studies. Consequently, the comparison among publications evaluating the real effect of glucosamine and chondroitin sulfate on synovial joint tissues and biochemical markers is challenging. The results of this study showed a positive cartilage response and biochemical modulation in approximately half of the articles evaluated. As for the rest of the parameters, these dietary supplements did not appear to adequately supress the subchondral bone changes, the synovial inflammation or the osteophyte formation. However, further experimental studies may be needed to evaluate the nutraceutical effect at those levels. Generally, beneficial effects were associated with a pre-emptive treatment administration, higher doses and multimodality approaches with some combined therapies. Even though some results were promising and encouraging, most of them continue to show a great heterogeneity and at present, there is a need to design high-quality systematic experimental studies. Additional studies focused on long-term treatments, as well as evaluating their potentially disease-modifying effects are required.

## Figures and Tables

**Table 1:** Main characteristics and results of preclinical animal studies of the effect of glucosamine and chondroitin sulfate in knee osteoarthritis.

References	Animal Model (*n*)	OA Model	Therapy: Dosage, Frequency and Administration Route (Duration Treatment)	Start Point ^(1)^	Follow-Up ^(2)^ Outcome Measures	Main Results
Abdul-Kadir et al. 2019 [33]	New Zealand Rabbit Male 7–8 months old (33)	Surgically induced OA: ACLT	GS (77.5 mg/kg/day) orally Channa (51.4 mg/kg/day) orally (8 weeks)	3 weeks	8 weeks Macroscopic evaluation. Histology of cartilage (modified OARSI score). Histomorphometry (cartilage roughness and Cg.Th). Serum biomarkers (COMP, COX-2 and PGE_2_)	Both treatment groups showed lower histopathology changes compared to the untreated group. However, Channa showed less cartilage roughness compared to GS treated-animals. Channa and GS significantly ↓COMP levels
Jeong et al. 2017 [34]	New Zealand Rabbit 9 month old (24)	Surgically induced OA: ACLT	GH (100 mg/kg/day), orally Celecoxib (10 mg/kg/day), orally MucoP (100 mg/kg/day), orally (8 weeks)	3 days	8 weeks X-ray. Macroscopic evaluation. Histology of cartilage. GAG quantification. TUNEL assay (Apoptosis of chondrocytes).	Macroscopically GH and MucoP groups had significantly milder cartilage damage and fibrillation. All the treatments investigated showed significantly reduced histology degenerative changes and prevented the apoptosis od chondrocytes.
Roman-Blas et al. 2017 [35]	New Zealand Rabbit Male (20)	Surgically induced OA: ACLT and partial MMT	CS (1200 mg/day) + GS (1500 mg/day), orally CS (1200 mg/day) + GH (1500 mg/day), orally (14 weeks)	−14 days	12 weeks Histology of cartilage (Mankin score) and synovial membrane (Krenn scale); X-ray densitometer scanner (Subchondral BMD) and Western blot studies (COX-2, IL-1β, iNOS, MMP-1, MMP-3, MMP-13)	Treatments did not significantly modify the cartilage damage or the synovial inflammation and failed to conserve the subchondral BMD. In addition, were unable to decrease the biochemical OA markers expression.
Permuy et al. 2015 [36]	New Zealand Rabbit Female Adult (56)	Surgically induced OA: ACLT and partial MMT	GS (21.5 mg/kg/day) orally CS (11.5 mg/kg/day) i.p. HA (0.3 mg/kg/week) i.a. DC (1.5 mg/kg/day) orally RIS (0.07 mg/kg/day) orally GS + RIS daily orally (8 weeks)	3 weeks	11 weeks Histology of cartilage and synovial membrane (OARSI score). Histomorphometric evaluation (Tb.A, Tb.Sp, SB.Th, FI, Cg.Th) and μ-CT (vBMD, BV/TV, Tb.Th, Tb.Sp, Tb.N, Tb.Pf, SMI, nCgTh)	GS and CS prevented cartilage swelling but did not reduce the cartilage damage or the superficial fibrillation. In addition, they did not have any effect on synovial and subchondral bone.
Ohnishi et al. 2013 [37]	Japanese Rabbit Female 12 weeks old (12)	Surgically induced OA: ACLT	FCP (1000 mg/day), orally GH (1000 mg/day), orally FCP + GS daily orally (4 weeks)	0 days	4 weeks Macroscopic evaluation. Histology of cartilage (modified Mankin score). IHQ (CTX-II). Serum biomarkers (CS-846, HA, KS)	Administration of FCP and/or GH showed chondroprotective effects. However, there were no significant differences in the biomarker concentrations comparing with untreated animals (↑CS846 and HA)
Wang et al. 2007 [38]	New Zealand Rabbit Male 9 month old (26)	Surgically induced OA: ACLT	GH (100 mg/day) orally (8 weeks)	1 day	8 weeks Macroscopic evaluation. X-ray absorptiometry scanner (BMD). Histomorphomethry (osteoid volumen, osteoid thicknes, BV/TV, Tb.Th, Tb.Sp, Tb.N, SB.Th)	GH tended to have lower severity of cartilage lesions but this difference was not significant. Additionally, GH prevented the subchondral bone changes and prevented the trabecular bone loss.
Kobayashi et al. 2005 [39]	Japanese Rabbit Male 13 weeks old (30)	Surgically induced OA: Partial MMT	GH (1000 mg/kg/day) + CS (800 mg/kg/day), orally GH + CS + Fursultiamine (100 mg/kg/day), orally (8 weeks)	3 days	8 weeks Macroscopic evaluation. Histology of cartilage (modified Mankin score). IHQ (MMP-1)	The GH + CS treatment slightly reduce the severity of cartilage lesions but no significantly. Only the group with received the combined treatment with fursultiamine showed a significant chondroprotective effects and ↓MMP-1.
Tiraloche et al. 2005 [40]	New Zealand Rabbit Male 9 month old (39)	Surgically induced OA: ACLT	GH (100 mg/day) orally (8 weeks)	3 weeks	11 weeks Macroscopic evaluation. Histology of cartilage (modified Mankin score). GAG quantification and total type II collagen.	GH administration tended to have lower macroscopic severity grades compared with placebo group, but it was not significant. Regarding histology parameters, GH did not prevent the cartilage erosion nor superficial fibrillation.
Torelli et al. 2005 [30]	Norflok Rabbit Female 2.5–3 monts old (40)	Physically induced OA: Immobilization (12 weeks)	CS (120 mg/week) s.c. (12 weeks)	0 days	12 weeks Histology of cartilage (hematoxylin-eosin, Masson trichrome and picrosirius red) and proteoglycan content.	CS did not reduce the histological changes such as cartilage fibrillation, chondrocyte disorganization and ↓proteoglycan, compared to untreated animals.
Salman et al. 2019 [31]	Abino Rat Male Adult (25)	Physically induced OA: Immobilization (6 weeks)	GS (40 mg/kg/day), orally RIS (0.2 mg/kg/day), orally GS + RIS daily orally (6 weeks)	0 days	6 weeks Histology of cartilage (modified Mankin score). Histomorphometry (Cg.Th, chondrocytes number). IHQ (type II collagen)	Both treatments improved the articular cartilage damage with the least degenerative changes in the group treated with a combination of both drugs. GS and RIS alone did not prevent the chondrocytes number decrease. ↑type II collagen.
Sun et al. 2018 [27]	Sprague Dawley Rat Male 5–6 weeks old (48)	Chemically induced OA: MIA	CSSB (50 mg/kg/day), orally CSSB (100 mg/kg/day), orally CSSB (200 mg/kg/day), orally CS (200 mg/kg/day), orally (1 month)	0 days	In-vivo paint and bearing test, daily 1 month Histology of cartilage (OARSI score). Synovial markers (IL-1β, TNF-alpha, PGE_2_ and NO). Western-blot (MMP-1, MMP-3 and TIMP-1)	Treated groups exhibited significant reduced histopathological cartilage changes, relieved joint pain and showed ↓IL1β, TNF-alpha, PGE_2_ and NO. Additionally, regulated the protein expression (↓MMP-1 and MMP-3 and ↑TIMP-1). Dose-dependent manner.
Wang et al. 2018 [41]	Sprague Dawley Rat (40)	Surgically induced OA: ACLT and MMT	GS (2, 5 or 10 mg/kg/day), intraperitonally (1 month)	0 days	1 month Histology of cartilage. IHQ (type II collagen). Synovial fluid inflammatory mediators (NO and IL1β). Western-blot (MMP-1, MMP-13). qPCR (TIMP-1)	Glucosamine treatment prevented cartilage degradation, up-regulated the levels of type II collagen and ↓MMP-1 and MMP-13 and ↑TIMP-1, in a dose-dependent manner.
Ren et al. 2017 [42]	Rat Male (24)	Surgically induced OA: ACLT	CSSM (25 mg/twice daily), orally CS (shark) (25 mg/twice daily), orally (6 weeks)	4 weeks	10 weeks Macroscopic evaluation. Histology of cartilage (Mankin score). Synovial fluid inflammatory mediators (IL-1β, TNF-alpha, PGE_2_). TUNNEL assay (Apoptosis of chondrocytes). Western-blot (MMP-1 and TIMP-1).	Treated groups showed chondroprotective effects by inhibiting the cartilage degradation and the apoptosis of chondrocytes. ↓IL-1β, TNF-alpha, PGE_2,_ ↓MMP-1 and ↑TIMP-1.
Sanches et al. 2017 [43]	Wistar Rat Male 8 weeks old (40)	Surgically induced OA: ACLT	CS (400 mg/kg) + GS (500 mg/kg) 3 days/week, orally CS + GS + photobiomodulation (29 days)	2 days	30 days Histology of cartilage (OARSI score). Histomorphometry (chondrocytes density and CgTh). IHQ (IL-1β, IL-10, type II collagen)	All treated groups showed lower degenerative histological changes and chondrocytes density. Animals treated with CS + GS + PBM showed significant ↓IL-1β and ↑IL-10 and type II collagen compared to untreated group.
Terencio et al. 2016 [44]	Wistar Rat Female 10 weeks old (45)	OVX + Surgically induced OA: ACLT (2 weeks post-OVX)	CS (140 mg/kg/day) + GH (175 mg/kg/day), orally (12 weeks)	−2 weeks	10 weeks Histology of cartilage and synovial membrane (OARSI score). Synovial fluid inflammatory mediators by ELISA (IL-1β, TNF-alpha) and radioimmunoassay (PGE_2_). Serum biomarkers (CTX-II, MMP-3, OPG, RANKL and osteocalcin). μ-CT (BV/TV, Tb.Th, Tb.N and vBMD)	OARSI scores of cartilage degradation were decreased in the treated group. CS and GH showed reduced levels of inflammatory mediators (↓IL-1β and TNF-alpha, CTX-II, MMP-3, OPG, RANKL) and a tendency to prevent the bone microstructural changes (↑vBMD) although, without statistical significance.
Panahafir et al. 2014 [45]	Sprague Dawley Rat 9 month old (27)	Surgically induced OA: KTI	Celecoxib (2.86 mg/kg/day), orally GH (192 mg/kg/day), orally (4, 8 or 12 weeks)	0 days	4, 8 or 12 weeks μ-CT and MRI. Histology of cartilage and synovial membrane (RAKSS score)	None of the treatments prevented cartilage loss, synovial inflammation or subchondral sclerosis. Additionally, GH failed to prevent the osteophyte formation
Lee et al. 2014 [28]	Wistar Rat Male 6 weeks old (50)	Chemically induced OA: MIA	GS (125 mg/kg) + CS (125 mg/kg), daily orally Deer bone extract (250 or 500 mg/kg/day), orally (50 days)	0 days	50 days μ-CT (BV/TV, Tb.Th, Tb.N and Tb.Sp)	Both treatments relieved the morphological bone changes. ↑BV/TV and Tb.Th, ↓Tb.Sp.
Wen et al. 2010 [46]	Wistar Rat Male 2 moth old (36)	Surgically induced OA: ACLT	GS (250 mg/kg/day/, orally (10 weeks)	5 weeks	3, 6, 9, 12, 15 and 18 weeks Allodynia and weight-bearing Macroscopic evaluation. Histology of cartilage and synovial membrane (Mankin score). IHQ (p38, JNK, ERK and MAPKs)	GS treated animals showed significantly lower cartilage damage and suppressed the synovial inflammation. Additionally, reduced the allodynia and weight bearing. ↓p38 and JNK, ↑ERK.
Naito et al. 2010 [47]	Sprague Dawley Rat Male 10 week old (18)	Surgically induced OA: ACLT	GH (1000 mg/kg/day), orally (8 weeks)	0 days	56 days Macroscopic evaluation. Histology of cartilage (Mankin score). Serum biomarkers (CTX-I, CTX-II, CPII)	GH administration suppressed the macroscopic changes and reduced the Mankin scores, but not significantly. ↓CTX-II, ↑CPII
Silva et al. 2009 [48]	Wistar Rat Male (?)	Surgically induced OA: ACLT	GS (500 mg/kg/day), orally GS (500 mg/kg/day) + CS (400 mg/kg/day), orally (70 days)	−7 days	In-vivo joint pain 70 days Histology of cartilage (OARSI score). Densitometry (CS content of cartilage)	GS + CS significantly prevented the cartilage histology alterations. Additionally, significantly reversed the increase in the CS cartilage quantification and reduced the joint pain.
Ivanovska et al. 2011 [29]	Outbred ICR (CD-2) Mice Male 10–12 weeks old (50)	Chemically induced OA: CIOA	GS (20 mg/kg/day), orally GH (20 mg/kg/day), orally (20 days)	0 or 7 days	30 days Histology of cartilage. Osteophyte area. Synovial fluid inflammatory mediators (RANKL, TNF-alpha, IL-6, IL-4 and IL-10). IHQ (RANKL, BMP-2)	GH significantly reduces the cartilage damage and osteophyte area. Additionally, ameliorates the OA progression by regulating the degree of bone resorption and bone remodeling. ↓RANKL, BMP-2 and IL-6, ↑IL-10.
Taniguchi et al. 2011 [32]	Hartley Guinea-pig Female 3 weeks old (50)	Spontaneusly model: Naturally occurring	GH (200 mg/kg/day), orally CS (200 mg/kg/day), orally (8, 12 or 18 months)	—	8, 12 or 18 months Histology of cartilage (modified Mankin score). TUNEL assay (Apoptosis of chondrocytes). mRNA levels for cartilage tissue (MMP-3, MMP-8, MMP-13, collagen type II and aggrecan)	Long-term GH or CS administration reduced the cartilage degeneration. Additionally, inhibited the loss of cartilage total RNA and the increase of MMP-3 mRNA

Abbreviations: ACLT, anterior cruciate ligament transection; BMD, bone mineral density; BV/TV, bone volume fraction; CgTh, cartilage thickness; cCgTh, calcified cartilage thickness; CIOA, collagenase induced osteoarthritis; μ-CT, micro-computed tomography; COMP, cartilage oligomeric matrix protein; COX, cyclooxygenase; CPII, type II collagen synthesis, CS, Chondroitin sulfate; CSSB, chondroitin sulfate from sturgeon bone; CSSM, chondroitin sulfate from scophthalmus maximus; CTX, collagen type I crosslinked C-telopeptide; CTX-II, collagen type II crosslinked C-telopeptide; DC, Diacerein; ERK, extracellular signal-regulated kinase; FCP, fish collagen peptide; FI, fibrillation index; GAG, glycosaminoglycans; GH, glucosamine hydrochloride; GlcN, glucosamine; GS, glucosamine sulfate; HA, hyaluronic acid; IHQ, immunohistochemistry; IL, Interleukin; iNOS, inducible nitric oxide synthase; JNK, c-Jun N-terminal kinase; KS, keratan sulfate; KTI, knee triad injury; MAPKs, mitogen-activated protein kinases; MIA, monosodium iodoacetate; MMP, metalloproteinase; MMT, medial meniscectomy; nCgTh, non-calcified cartilage thickness; NO, nitric oxide; OARSI, Osteoarthritis Research Society International; OPG, osteoprotegerin; OVX, ovariectomized; PG, prostaglandin; qPCR, quantitative polymerase chain reaction; RAKSS; rat arthritis knee scoring system; RANKL, receptor activator of nuclear factor-kappa B ligand; RIS, risedronate; SB.Th, subchondral bone plate thickness, SMI structural model index; Tb.A, trabecular area, Tb.N trabecular number; Tb.Pf trabecular bone pattern factor, Tb.S, trabecular separation; Tb.Th, trabecular thickness; TIMP, tissue inhibitor of metalloproteinases; TNF, tumor necrosis factor; TUNEL, terminal deoxynucleotidyl transferase-mediated nick-end labeling; ↑, increase; ↓, decrease. ^(1)^ Start point: time between induced OA and treatment administration. ^(2)^ Follow-Up: time between induced OA and the evaluations carried out in the study.

**Table 2:** Synthesis of main outcomes of the effect of nutraceuticals.

Nutraceutical	Reference	Initial Adminst Ration	C	SB	SM	OST	BM
Glucosamine sulfate (GS)	Abdul-Kadir et al. [33]	Delayed	+	x	x	x	+
*n* = 6	Permuy et al. [36]	Delayed	−	−	−	x	x
	Salman et al. [31]	Early	+	x	x	x	+
	Wang et al. [41]	Early	+	x	x	x	+
	Wen et al. [46]	Delayed	+	x	+	x	+
	Ivanovska et al. [29]	Early	-	x	x	-	-
Glucosamine hydrochloride (GH)	Jeong et al. [34]	Early	+	x	x	x	+
*n* = 8	Ohnishi et al. [37]	Early	+	x	x	x	−
	Wang et al. [38]	Early	?	+	x	x	x
	Tiraloche et al. [40]	Delayed	?	x	x	x	−
	Panahafir et al. [45]	Early	-	-	-	-	x
	Naito et al. [47]	Early	?	x	x	x	+
	Ivanovska et al. [29]	Early	+	x	x	+	+
	Taniguchi et al. [32]	Pre-emptive	+	x	x	x	+
Chondroitin sulfate	Permuy et al. [36]	Delayed	−	−	−	x	x
*n* = 5	Torelli et al. [30]	Early	−	x	x	x	x
	Sun et al. [27]	Early	+	x	x	x	+
	Ren et al. [42]	Delayed	+	x	x	x	+
	Taniguchi et al. [32]	Pre-emptive	+	x	x	x	+
Chondroitin sulfate + GH	Roman-Blas et al. [35]	Pre-emptive	−	−	−	x	−
*n* = 3	Kobayashi et al. [39]	Early	?	x	x	x	?
	Terencio et al. [44]	Pre-emptive	+	?	?	x	+
Chondroitin sulfate + GS	Roman-Blas et al. [35]	Pre-emptive	−	−	−	x	−
*n* = 4	Sanches et al. [43]	Early	+	x	x	x	?
	Lee et al. [28]	Early	x	+	x	x	x
	Silva et al. [48]	Pre-emptive	+	x	x	x x	+

C cartilage, SB subchondral bone, SM synovial membrane, OST osteophyte, BM biochemical markers. (+) Positive effect; (−) negative effect or no effect; (?) unclear or not significantly effect; (x) not included. Therapy initial administration: Pre-emptive (before OA induction), early (OA induction- 14 days post); delayed (>14 days post-OA induction).

**Table 3:** Therapy duration of nutraceuticals.

Animal Model	Short-term (≤8 Weeks)			Intermediate-Term (>8 to <24 Weeks)			Long-Term (≥24 Weeks)		
	Reference	Therapy	Duration	Reference	Therapy	Duration	Reference	Therapy	Duration
Rabbit	Abdul-Kadir et al. [33]	GS	8 weeks	Roman-Blas et al. [35]	GH/GS + CS	14 weeks			
	Jeong et al. [34]	GH	8 weeks	Torelli et al. [30]	CS	12 weeks			
	Permuy et al. [36]	GS/CS	8 weeks						
	Ohnishi et al. [37]	GH	4 weeks						
	Wang et al. [38]	GH	8 weeks						
	Kobayashi et al. [39]	GH + CS	8 weeks						
	Tiraloche et al. [40]	GH	8 weeks						
Rat	Salman et al. [31]	GS	6 weeks	Terencio et al. [44]	GH + CS	12 weeks			
	Sun et al. [27]	CS	4 weeks	Panahafir et al. [45]	GH	12 weeks			
	Wang et al. [41]	GS	4 weeks	Wen et al. [46]	GS	10 weeks			
	Ren et al. [42]	CS	6 weeks	Silva et al. [48]	GS + CS	10 weeks			
	Sanches et al. [43]	GH + CS	4 weeks						
	Lee et al. [28]	GS + CS	7 weeks						
	Naito et al. [47]	GH	8 weeks						
Mice	Ivanovska et al. [29]	GH /GS	3 weeks						
Guinea-Pig							Taniguchi et al. [32]	GH/CS	18 months

Abbreviations: CS, Chondroitin sulfate; GH, glucosamine hydrochloride; GS, Glucosamine sulfate.

## Data Availability

Not applicable.
